# Nominal-Model-Based Sliding-Mode Control for Traveling-Wave Ultrasonic Motor

**DOI:** 10.3390/mi13111846

**Published:** 2022-10-28

**Authors:** Jing Liang, Kai Jing, Yan Dong, Xiaping Lin, Yuqing Wang

**Affiliations:** 1 School of Artificial Intelligence, Hebei University of Technology, Tianjin 300130, China; 2 State Key Laboratory of Reliability and Intelligence of Electrical Equipment, Hebei University of Technology, Tianjin 300130, China; 3 School of Electrical and Engineering, Hebei University of Technology, Tianjin 300130, China; 4 Fujian Branch of China Mobile Communication Group Co., Ltd., Fuzhou 350001, China

**Keywords:** ultrasonic motor, piezoelectric effect, sliding-mode control

## Abstract

Traveling-wave ultrasonic motors (TWUSMs) have strong nonlinearity and uncertainty, which are sensitive to the environment, disturbances, and load changes. Thus, precision control of TWUSMs is hard to achieve with traditional methods for complex driving mechanisms. A nominal-model-based sliding-mode control strategy with strong robustness is proposed to achieve accurate speed control of TWUSMs. Firstly, a second-order nominal model of the speed difference and output torque was deduced to construct a nonlinear sliding-mode surface; then, a nonlinear sliding-mode controller was designed with the collaborative regulation of frequency and the amplitude of two-phase control voltages. The global asymptotic stability of the controller was proved under bounded disturbances and parameter uncertainty. Finally, the effectiveness and accurate control were testified to and verified by the simulations and experiments, which showed good robustness and a disturbance rejection of the strategy for TWUSMs, with strong nonlinearity and uncertainty.

## 1. Introduction

A TWUSM is a typical piezoelectric motor with the advantages of having high torque at low speeds, excellent start/stop dynamics, and a simple mechanical design, as well as being non-electromagnetic, which is suitable for precision driving systems in the medical and aerospace fields, among others [1,2,3,4,5]. However, the disadvantages of nonlinearity and parameter uncertainty introduced by inverse piezoelectric effect and contact frictions seriously affect control accuracy and increase the difficulty of control [6,7,8,9].

In order to obtain a better speed-control performance, some robust control methods have been investigated. The traditional PID control method with natural robustness has been modified to adapt to the control of TWUSMs. In Ref. [10], a variable-gain-type IMC-PID, combined with the neural network, was proposed, which uses the neural network to compensate for the nonlinearity of TWUSMs. In Ref. [11], a PSO-based PID speed-control algorithm was designed to optimize the controller parameters in real-time to solve the time-varying parameters caused by temperature changes. In Ref. [12], an algorithm that combines pattern reasoning with PID to achieve speed control was proposed. In Ref. [13], a variable-gain PID controller based on a neural network was designed. In Ref. [14], an online adjustment method of PID parameters with two expert rules was put forward, which overcame the nonlinearity and load disturbance of TWUSMs. Although the structure of the PID controller is simple, it is more suitable for linear systems instead for nonlinear systems, such as TWUSMs. Achieving fast and accurate dynamic control remains challenging, although PID parameters can be self-tuned.

In addition, some researchers focus on intelligent learning algorithms for a low dependence on the parameters. A speed controller was designed with a neural network inverse model of the TWUSM in [15], which used the error backpropagation algorithm through on-line learning to achieve compensation for the characteristic changes of TWUSMs to reduce the time cost. In Ref. [16], a speed-control method based on nonlinear iterative-learning control law was proposed. A secant iterative-learning method was proposed in [17] to overcome the difficulty of determining the differential term of the Newton iterative-learning method. However, the iterative-learning method is mainly used in repetitive motion control and is weak in robustness against non-periodic disturbances.

Moreover, the sliding-mode control (SMC) has attracted more attention for its no accurate dynamic model requirements and strong robustness. In Ref. [18], a sliding-mode control method with two inputs, namely the frequency and phase difference, was designed to achieve the position control of TWUSMs. Alem et al. [19] designed an adaptive sliding-mode control based on a parameter estimator and parameter adaptive rules. However, for the traditional SMC, the strong robustness only exists in the sliding stage and not in the arrival stage, which will lead to a high gain risk to bring in the overshoot of the controller [20]. A nominal-model-based sliding-mode control (NMSMC) will weaken the above risk and keep the robustness in both the arrival stage and the sliding stage by the nominal model response to structure a nonlinear sliding surface [21].

In this paper, a nominal-model-based sliding-mode control method was studied to improve the speed, accuracy, and robustness of TWUSMs. Firstly, the approximately linear relationship between speed difference and output torque was deduced from the mechanical characteristics of the TWUSM to construct a second-order nominal model. Furthermore, a traditional linear regulator was designed based on the nominal model to obtain a good response. Secondly, a nonlinear SMC was designed based on the nonlinear sliding-mode surface constructed by the dynamic response of the nominal model, which enhances global robustness. Furthermore, the amplitude and frequency of the two-phase voltages were collaboratively regulated in the controller to achieve a better effect of speed response sectionally. The global asymptotic stability and robustness of the control were proved under external disturbances and parameter uncertainties. Finally, the effectiveness was verified and testified by simulations and experiments.

The rest of the paper is organized as follows. In Section 2, the mechanism and simplified speed model of TWUSMs are presented. Section 3 presents the NMSMC design of TWUSMs. Section 4 presents the simulations and experiments of the proposed controller under different working conditions. Section 5 provides the conclusions.

## 2. Modeling of TWUSMs

### 2.1. Mechanism of TWUSMs

**Figure 1:**
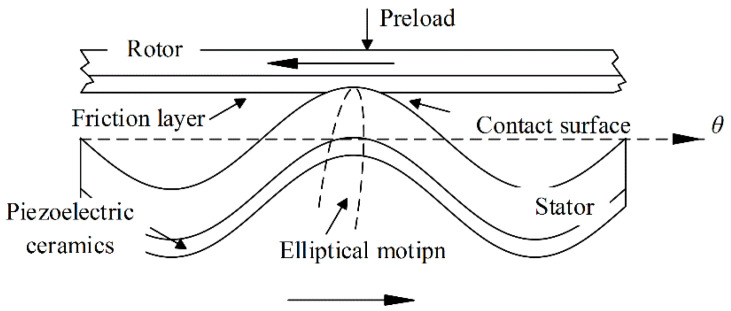
Contact surface between stator and rotor.

As shown in Figure 1, a TWUSM mainly consists of a stator with piezoelectric ceramics on the bottom and a rotor with a friction layer. With the inverse piezoelectric effect, the stator excites a traveling-wave vibration by supplying two-phase sinusoidal driving voltages with an equal amplitude and π/2 phase difference. The traveling wave $w\left(t\right)$ can be expressed as:

(1) $$w\left(t\right)=w_{A}\cos\left(k\theta \right)+w_{B}\sin\left(k\theta \right)=W\sin(2\pi ft)\cos(k\theta )+W\cos(2\pi ft)\sin(k\theta ),$$

where $w_{A}=W\sin(2\pi ft)$ and $w_{B}=W\cos(2\pi ft)$, which represent the two-phase vibration mode, and f is the frequency of the traveling wave. W is the amplitude of the vibration mode and k is the wavenumber of the traveling wave on the stator surface.

For the rotor, the friction layer is pressed on the surface of the stator by preloading, and the rotor is driven by friction force, which is generated through the elliptical motions of the particles on the stator surface, excited by the traveling wave. The driving torque $T_{d}$ generated by the circumferential friction force $F_{\theta }$ can be expressed as [22]:

(2) $$T_{d}=R_{w}F_{\theta }=R_{w}k\int_{-\theta_{0}}^{\theta_{0}}\xi_{\theta }\mu p\left(\theta \right)d\theta =R_{w}k\int_{-\theta_{0}}^{\theta_{0}}\frac{v_{s\theta }-v_{R}}{\sqrt{{\left(v_{s\theta }-v_{R}\right)}^{2}+v_{\mathrm{sr}}^{2}}}\mu p\left(\theta \right)d\theta ,$$

where $R_{w}$ is the contact radius; $\xi_{\theta }$ is the circumferential coefficient; $\theta_{0}$ is the contact area boundary of the stator and rotor; $v_{s\theta }$ and $v_{\mathrm{sr}}$ are the circumferential and radial speed of the stator particle, respectively; $v_{R}$ is the speed of the rotor; μ is the friction coefficient; and $p\left(\theta \right)$ is the normal contact pressure distribution function. The formula of the rotational motion is calculated by (3), according to the balance of the forces.

(3) $$I_{r}{\ddot{\theta }}_{r}+C_{r}{\dot{\theta }}_{r}=T_{d}-T_{L}-d,$$

where $\theta_{r}$ is the rotor position; $I_{r}$ is the rotor inertia; $C_{r}$ is rotor damping; d is the external disturbances; and $T_{d}$ and $T_{L}$ are the driving torque and load torque, respectively.

### 2.2. Simplified Speed Model of TWUSMs

The mechanism model above is not suitable for the controller design due to the complex process of the vibration and contact friction of TWUSMs. Therefore, some researchers proposed an approximate expression, as shown in (4), to describe the relationship between the speed difference and output torque [18,23,24].

(4) $$T_{d}=f_{r}\left(\omega_{s}-{\dot{\theta }}_{r}\right),$$

where $\omega_{s}$ is the maximum circumferential stator angular speed; ${\dot{\theta }}_{r}$ is the rotor angular speed; and $f_{r}$ is the model coefficient.

**Figure 2:**
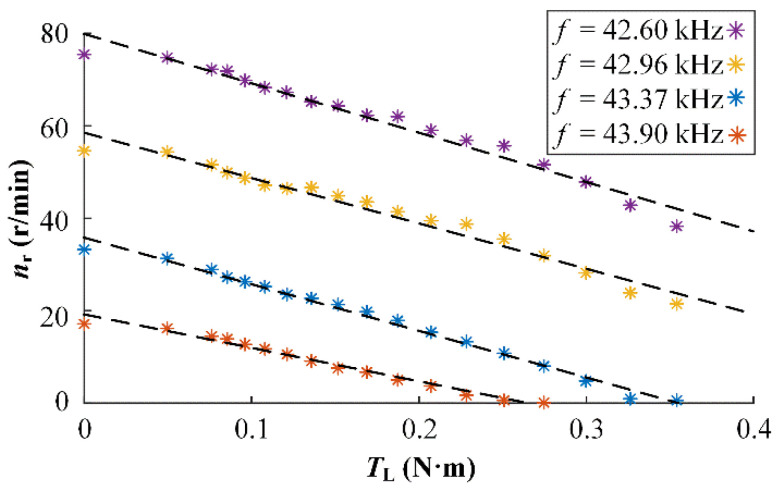
Rotor revolving speed vs. load at constant input.

To verify the relationship, $T_{L}$ and $n_{r}$ at different f were tested experimentally, and the mechanical characteristic curves shown in Figure 2 were obtained. The relationship between $T_{L}$ (=$T_{d}$, uniform motion) and rotor speed $n_{r}$ (=30 ${\dot{\theta }}_{r}/\pi$) under the same stator angular speed $\omega_{s}$ is almost linear for each frequency, which is fitted as the dashed line. However, the slope of the curve representing the model coefficient $f_{r}$ above does not remain constant, and the speed difference is nearly zero when $T_{L}$ = $T_{d}$ = 0 by analyzing the Formula (2).

Therefore, uncertainty and variability still exist in the model, which affect control accuracy, although models (3) and (4) can reduce the expression complexity of TWUSMs.

## 3. Controller Design

To solve the problems above, a nominal-model-based sliding-mode nonlinear controller is more suitable for the speed control of TWUSMs. It can achieve strong robustness in both the arrival and sliding stages by constructing a nonlinear surface based on an inaccurate nominal model.

### 3.1. Linear Controller for the Nominal Model

According to (3) and (4), a practical second-order model is established and expressed as:

(5) $$G_{r}{\ddot{\theta }}_{r}+F_{r}{\dot{\theta }}_{r}=\omega_{s}-D,$$

where $G_{r}=\frac{I_{r}}{f_{r}}$, $F_{r}=\frac{C_{r}}{f_{r}}+1$, and $D=\frac{T_{L}+d}{f_{r}}$.

Under the premise of bounded variation, a nominal model with constant parameters and no disturbances can be obtained, although there are parameter uncertainties and ex-ternal disturbances in the system, as shown in the following formula:

(6) $$G_{n}{\ddot{\theta }}_{n}+F_{n}{\dot{\theta }}_{n}=\omega_{n},$$

where $G_{n}$ and $F_{n}$ are the nominal coefficients, ${\dot{\theta }}_{n}$ is the nominal rotor speed, and $\omega_{n}$ is the control input of the nominal model.

A linear controller is designed based on the error for the linear model (6). The desired speed is set to ${\dot{\theta }}_{d}$, then the speed error of the nominal model is defined as ${\dot{e}}_{1}={\dot{\theta }}_{n}-{\dot{\theta }}_{d}$. ${\dot{e}}_{1}$ can be substituted into (6), expressed as follows:

(7) $$G_{n}({\ddot{e}}_{1}+{\ddot{\theta }}_{d})+F_{n}({\dot{e}}_{1}+{\dot{\theta }}_{d})=\omega_{n}.$$

The control law for the nominal model is designed as:

(8) $$\omega_{n}=G_{n}(-h_{1}e_{1}-h_{2}{\dot{e}}_{1}+\frac{F_{n}}{G_{n}}{\dot{\theta }}_{d}+{\ddot{\theta }}_{d}).$$

Using the control law (8), (9) is gained.

(9) $${\ddot{e}}_{1}+\left[\frac{F_{n}}{G_{n}}+h_{2}\right]{\dot{e}}_{1}+h_{1}e_{1}=0.$$

In order to guarantee the stability of the system, $h_{1}$ and $h_{2}$ need to make all the solutions of the Hurwitz Formula (10) have a negative real part, in which σ is Laplacian.

(10) $$\sigma^{2}+\left[\frac{F_{n}}{G_{n}}+h_{2}\right]\sigma +h_{1}=0.$$

### 3.2. Sliding-Mode Controller on the Nominal Model

A practical plant is described as (5), in which the parameters meet the following conditions: $G_{m}$ < $G_{r}$ < $G_{M}$, $F_{m}$ < $F_{r}$ < $F_{M}$, and D ≤ $D_{m}$. As such, the SMC is designed based on the response of the nominal model controlled by the linear controller.

The position error between the nominal model and the practical plant can be defined as $e_{2}=\left(\theta_{r}-\theta_{n}\right)$. The sliding surface is designed as follows:

(11) $$s={\dot{e}}_{2}+\lambda e_{2},$$

where $\lambda =F_{n}/G_{n}>0$.

In order to maintain *s* = 0, the global sliding-mode control law is introduced as (12). Define the medium values $G_{a}=\left(G_{m}+G_{M}\right)/2$ and $F_{a}=\left(F_{m}+F_{M}\right)/2$.

(12) $$\omega_{s}=-Ks-h\mathrm{sgn}(s)+G_{a}(\frac{1}{G_{n}}\omega_{n}-\lambda {\dot{\theta }}_{r})+F_{a}{\dot{\theta }}_{r},$$

where K>0. The control law and stability are proved in the next part.

### 3.3. Stability Analysis

To prove the global asymptotic uniform stability under the bounded disturbances and parameter deviation, the Lyapunov function is selected as:

(13) $$V=\frac{1}{2}G_{r}s^{2}.$$

The derivative with respect to time is:

(14) $$\begin{array}{ll} \dot{V} & =sG_{r}\dot{s}=sG_{r}\left[\left({\ddot{\theta }}_{r}-{\ddot{\theta }}_{n})+\lambda ({\dot{\theta }}_{r}-{\dot{\theta }}_{n}\right)\right]\\ & =s[(G_{r}{\ddot{\theta }}_{r}+F_{r}{\dot{\theta }}_{r})-\frac{G_{r}}{G_{n}}(G_{n}{\ddot{\theta }}_{n}+F_{n}{\dot{\theta }}_{n})-F_{r}{\dot{\theta }}_{r}+G_{r}\lambda {\dot{\theta }}_{r}]\\ & =s\left[\omega_{s}-D-\frac{G_{r}}{G_{n}}\omega_{n}-F_{r}{\dot{\theta }}_{r}+G_{r}\lambda {\dot{\theta }}_{r}\right] \end{array}$$

By substituting the control law (12) into (14), $\dot{V}$ becomes:

(15) $$\begin{array}{ll} \dot{V} & =s\left[-Ks-h\mathrm{sgn}\left(s\right)+G_{a}\left(\frac{1}{G_{n}}\omega_{n}-\lambda {\dot{\theta }}_{r}\right)+F_{a}{\dot{\theta }}_{r}-D-\frac{1}{G_{n}}G_{r}\omega_{n}-F_{r}{\dot{\theta }}_{r}+G_{r}\lambda {\dot{\theta }}_{r}\right]\\ & =-Ks^{2}-h\left|s\right|+s\left[\left(G_{a}-G_{r}\right)\left(\frac{1}{G_{n}}\omega_{n}-\lambda {\dot{\theta }}_{r}\right)+\left(F_{a}-F_{r}\right){\dot{\theta }}_{r}-D\right]\\ & \leq -Ks^{2}-h\left|s\right|+\left|s\right|\left[\left|G_{a}-G_{r}\right|\left|\frac{1}{G_{n}}\omega_{n}-\lambda {\dot{\theta }}_{r}\right|+\left|F_{a}-F_{r}\right|\left|{\dot{\theta }}_{r}\right|+\left|D\right|\right] \end{array}$$

According to the definition of the medium values of $G_{a}$ and $F_{a}$, the following formula can be obtained:

(16) $$\left\{\begin{array}{l} \frac{1}{2}\left(G_{M}-G_{m}\right)\geq \left|G_{a}-G_{r}\right|\\ \frac{1}{2}\left(F_{M}-F_{m}\right)\geq \left|F_{a}-F_{r}\right| \end{array}\right..$$

So, when *h* satisfies:

(17) $$h\geq \frac{1}{2}\left(G_{M}-G_{m}\right)\left|\frac{1}{G_{n}}\omega_{d}-\lambda {\dot{\theta }}_{r}\right|+\frac{1}{2}\left(F_{M}-F_{m}\right)\left|{\dot{\theta }}_{r}\right|+\left|D_{M}\right|,$$

there must be

(18) $$\dot{V}\leq -Ks^{2},$$

which means that the Lyapunov function *V* is an exponential convergence.

In addition, the sliding surface *s*, which can be written as (19), is a nonlinear dynamic function. Consequently, the NMSMC has global robustness and avoids high gains risk.

(19) $$s=\left({\dot{\theta }}_{r}-{\dot{\theta }}_{d}\right)+\lambda \left(\theta_{r}-\theta_{d}\right)-\left({\dot{e}}_{1}+\lambda e_{1}\right).$$

The sign function sgn(·) in (12) can be replaced with a sigmoid function to reduce chattering.

### 3.4. Control Law of Amplitude-Frequency Coordination

The control quantity $\omega_{s}$ can be adjusted by the amplitude $U_{\mathrm{amp}}$, frequency *f*, and phase difference φ of the two-phase control voltages [25,26,27]. Approximately, $\omega_{s}$ can be expressed by the following formula when φ=π/2 [28].

(20) $$\omega_{s}=m_{1}U_{\mathrm{amp}}e^{n_{1}+n_{2}f}.$$

In practice, considering the dead zone phenomenon caused by the inverse piezoelectric effect and contact friction [29], the relationship can be rewritten as:

(21) $$\omega_{s}=(m_{1}U_{\mathrm{amp}}-m_{2})e^{n_{1}+n_{2}f},U_{\mathrm{amp}}>U_{\mathrm{th}},$$

where $m_{1}$, $m_{2}$, $n_{1}$, and $n_{2}$ are viewed as constants, but are related to loading and temperature, etc.

**Figure 3:**
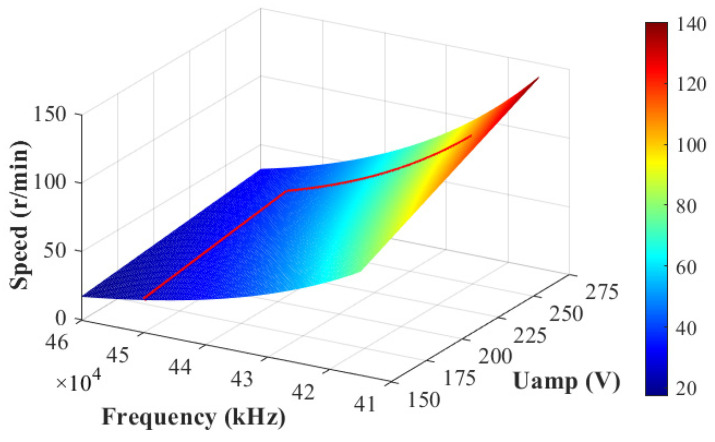
TWUSM speed, depending on the amplitude of voltage and frequency.

In this paper, sectional control is adopted. That is, $\omega_{s}$ is adjusted only by $U_{\mathrm{amp}}$ with the maximum frequency $f_{\max}$ when $\omega_{s}\leq \omega_{m}$ and by *f* with the maximum $U_{\mathrm{amp}}$ when $\omega_{s}>\omega_{m}$. For the characteristic of $U_{\mathrm{amp}}$-*f*-$\omega_{s}$ of a GTUSM-60-R-typed TWUSM, which is shown in Figure 3, the control section can be summarized as:

(22) $$\left\{\begin{array}{l} \left\{\begin{array}{l} U_{\mathrm{amp}}=\frac{\left(9.5492\omega_{s}+m_{2}\right)}{m_{1}}\\ f=f_{\max}=45\mathrm{kHz}\; \end{array}\right.,\;\mathrm{when}\;\omega_{s}\leq \omega_{m}\\ \left\{\begin{array}{l} U_{\mathrm{amp}}=250V\;\\ f=\frac{\left(\ln\left(\omega_{s}\right)+2.2565-n_{1}\right)}{n_{2}} \end{array}\right.,\;\mathrm{when}\;\omega_{s}>\omega_{m}\; \end{array}\right.,U_{\mathrm{amp}}>U_{\mathrm{th}},$$

As shown in Figure 3, the speed regulation curve is designed as the red line. The results of parameters identification are $m_{1}$ = 0.1547, $m_{2}$ = 2.83, $n_{1}$ = 18.47, and $n_{2}$ = 0.331.

**Figure 4:**
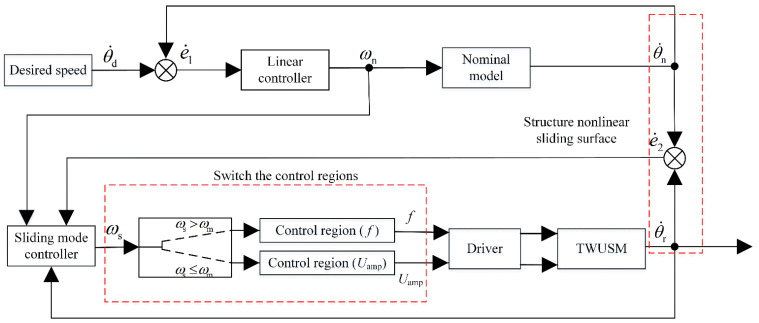
Block diagram of NMSMC for TWUSM.

The above design process is summarized in Figure 4.

## 4. Simulations and Experiments

In order to verify and testify to the efficiency of the control strategy, simulations, and experiments are carried out based on a GTUSM-60-R-typed TWUSM, of which the parameters are listed in Table 1. The value ranges of $C_{r}$ and $f_{r}$ are [$C_{m}$, $C_{M}$] and [$f_{m}$, $f_{M}$], respectively. The voltage amplitude and frequency adjusting range are [150 V, 250 V] and [42 kHz, 45 kHz], respectively. In addition, a traditional SMC was also tested as a comparison.

### 4.1. Dynamic Response of Nominal Model

**Figure 5:**
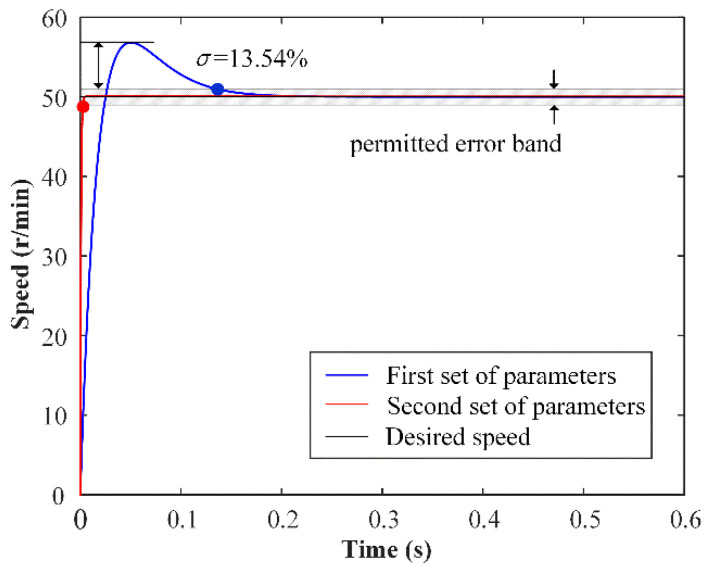
Nominal model step response for different parameters.

The nominal model response is related to the TWUSM performance because the nonlinear sliding surface *s* is constructed based on the response of the nominal model. There are two sets of parameters for two simulations, where the first set is $h_{1}$ = 1600, $h_{2}$ = −2.47 × 10^5^, and the second set is $h_{1}$ =1 × 10^4^, $h_{2}$ = −2.46 × 10^5^. The desired speed is 50 r/min. For the first set of parameters, the step response of the nominal model is the blue line in Figure 5. The speed of the nominal model reaches the permitted error band in 0.1353 s, which is ±2% of the desired speed, and the overshoot is 13.6%. For the second set of parameters, the step response of the nominal model is the red line. The speed of the nominal model almost reaches the permitted error band in the beginning, and there is no overshoot. In this paper, the second set of parameters is selected.

### 4.2. Simulation for Speed Control on NMSMC

**Figure 6:**
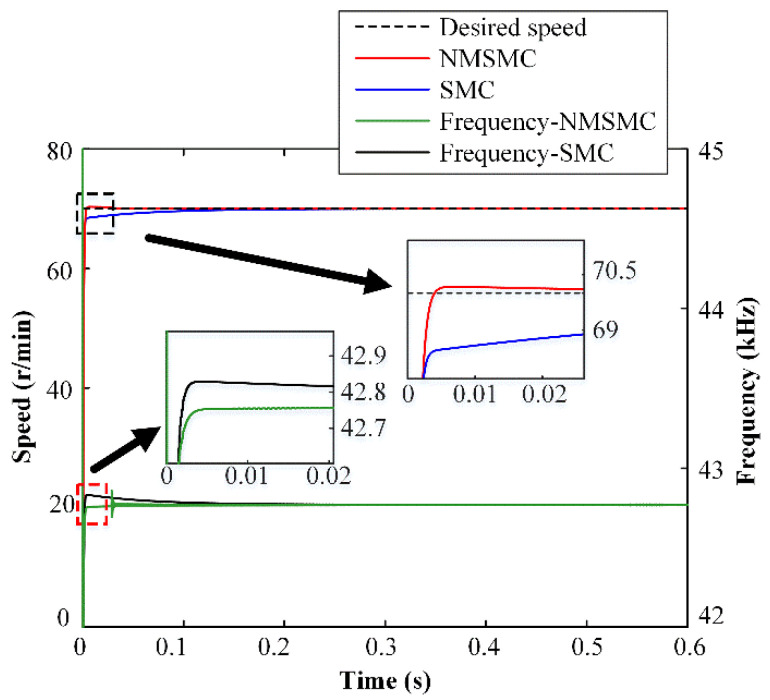
Simulation results of constant speed control for SMC and NMSMC.

The speed step responses of the TWUSM with the SMC and the NMSMC at the desired speed of 70 r/min under no-load conditions are shown in Figure 6. As a whole, both controllers reproduce the desired speed without significant deviations. However, the NMSMC reduces the rise time compared to the SMC, as shown in the zoomed part of the black dotted box. Moreover, the SMC reaches the control domain *f*, and the NMSMC control input is changed from $U_{\mathrm{amp}}$ to *f* to avoid high gain risk at the beginning, as shown in the zoomed part of the red dotted box. Furthermore, the NMSMC takes less time to reach the final stable *f* of 42.77 kHz than the SMC.

**Figure 7:**
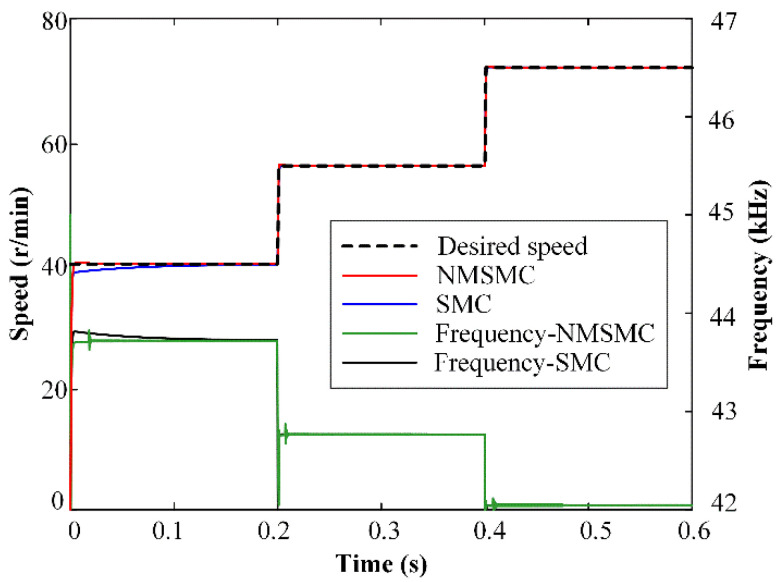
Simulation results of varying speed control for SMC and NMSMC.

The speed-tracking performances of the TWUSM with the SMC and the NMSMC are shown in Figure 7, when the desired speed is set from 50 to 70 to 90 r/min without load. With the desired speed changing, both controllers respond quickly, and there are no significant deviations in speed curves.

**Figure 8:**
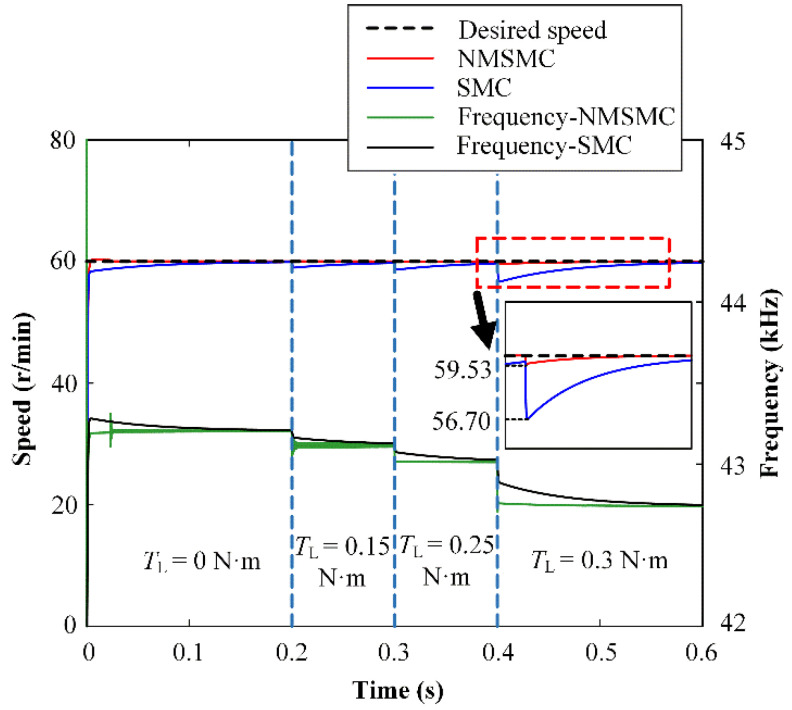
Simulation results of anti-load speed control for SMC and NMSMC.

The anti-load-disturbance abilities of the TWUSM under the control of the SMC and the NMSMC are shown in Figure 8 when the desired speed is set to 60 r/min, and the load $T_{L}$ is changed from 0 N·m to 0.3 N·m. The NMSMC can accurately track the desired speed, while the SMC is significantly disturbed when the load disturbance increases from 0 N·m to 0.25 N·m. Then, the NMSMC deviates from the desired speed to 59.53 r/min while the SMC deviates from the desired speed to 56.70 r/min when the load disturbance further increases to 0.3 Nm. The NMSMC does show a significant advantage in anti-load disturbance compared with the SMC.

It can be observed that there are some short-time oscillations in the frequency curves of Figure 6, Figure 7 and Figure 8, which are caused by the sliding-mode surface constructed by the dynamic response of the linear controller. In order to improve the rapidity, a reasonable overshoot is retained in the parameter design of the linear controller, which leads to oscillations of the frequency curves in the NMSMC. However, the rapidity and robustness of the velocity tracking are effectively guaranteed compared with the traditional SMC.

### 4.3. Experiments

**Figure 9:**
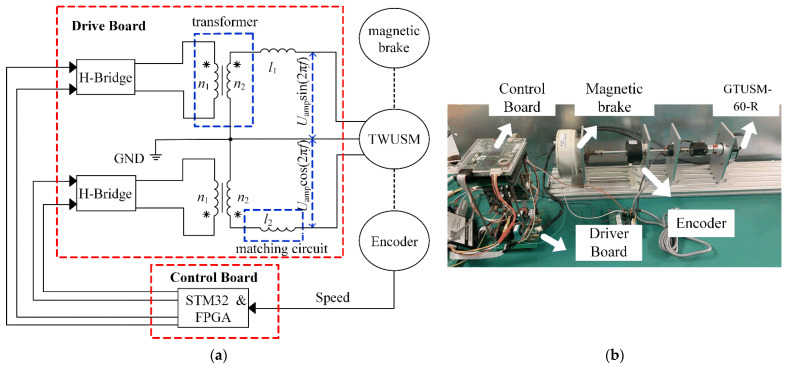
Experimental setup of the TWUSM system: (**a**) Configuration of experimental test setup; (**b**) experimental platform.

The NMSMC is also verified by experiments, and the experimental setup of the TWUSM drive system is shown in Figure 9. The drive board includes H bridges, transformers ($n_{1}$/$n_{2}$ is the transformer ratio), and matching circuits ($l_{1}$ and $l_{2}$ are inductance values). The control board comprises STM32 and FPGA. The encoder (YJK6010-G-2500BM-5L) can measure 2500 pulses/round in quadrature. The magnetic brake of type ZKB-0.3YN from Mitsubishi can generate a maximum torque of 3 N·m. With the aid of the STM32, the amplitude and frequency of driving voltages can be adjusted.

**Figure 10:**
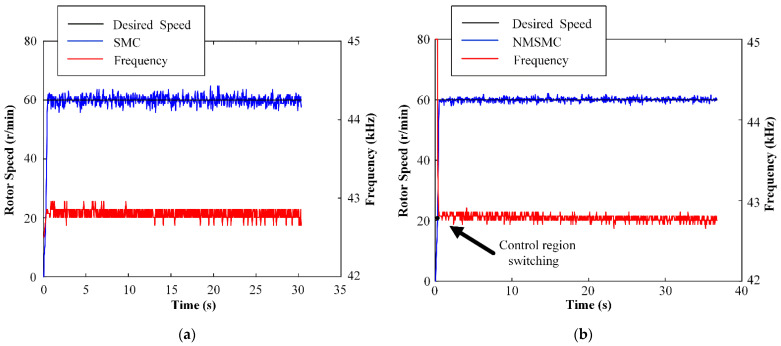
Experiment results of constant speed control: (**a**) SMC; (**b**) NMSMC.

The speed step responses and control inputs *f* of the TWUSM with the SMC and the NMSMC are shown in Figure 10 when the desired speed is set to 60 r/min and $T_{L}$ is 0 N·m. Overall, the rise times of the two controllers are similar, and both reproduce the desired speed. Detailed analysis shows that the speed error range of the SMC is from 4.20 r/min to 4.80 r/min, while the speed error range of the NMSMC is from 1.92 r/min to 2.04 r/min. Moreover, the SMC makes the input enter the control domain *f* at the beginning while the NMSMC controls the input to slide from $U_{\mathrm{amp}}$ to *f* to avoid the risk of high gain.

**Figure 11:**
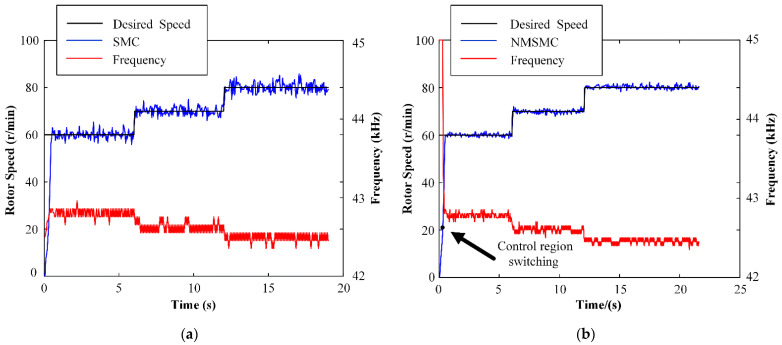
Experiment results of variable speed control: (**a**) SMC; (**b**) NMSMC.

The speed-tracking performances and control inputs *f* of the TWUSM with the SMC and the NMSMC are shown in Figure 11 when the desired speed changes from 60 to 70 to 80 r/min and $T_{L}$ = 0 N·m. Both controllers can respond to the desired speed quickly. Analyzing in detail, the speed error range of the SMC is from 4.40 r/min to 5.20 r/min, and the speed error range of the NMSMC is from −1.76 r/min to 1.84 r/min. Overall, the speed con-trolled by the NMSMC is smoother than that of the SMC.

**Figure 12:**
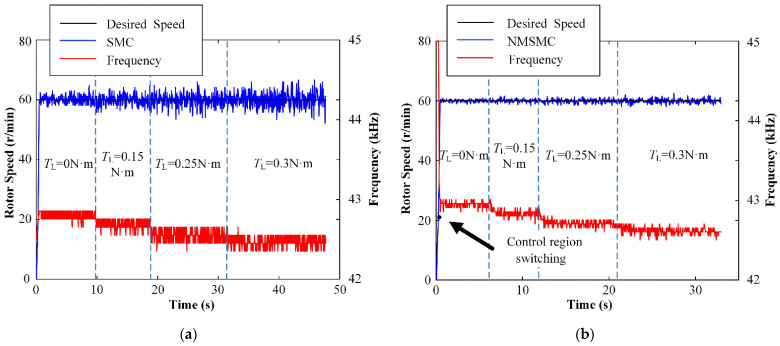
Experiment results of rotor-speed control when the load is applied: (**a**) SMC; (**b**) NMSMC.

The anti-load-disturbance abilities and control inputs *f* of the TWUSM with the SMC and the NMSMC are shown in Figure 12 when the desired speed is set to 60 r/min, and the load $T_{L}$ changes from 0 N·m to 0.3 N·m. It can be seen that both controllers can resist the disturbance of the load by adjusting *f*. Specifically, the speed error range of the SMC and the NMSMC are from −7.80 r/min to 6.80 r/min and from 2.04 r/min to 2.52 r/min. The anti-load-disturbance ability of the NMSMC has a significant advantage compared with the SMC.

Through the experiments above, with the changes in the desired speed and load, the speed-control performance of the NMSMC is significantly better than that of the SMC.

## 5. Conclusions

This paper proposed a nominal-model-based sliding-mode controller for the TWUSM to regulate speed by adjusting the frequency and amplitude of the two-phase control voltages. The controller used the linear control response of the second-order nominal model of the TWUSM as the nonlinear sliding-mode surface, which dramatically reduces the complexity of the mechanism model analysis of the TWUSM, but also introduces the necessary nonlinearity for the controller. The NMSMC of TWUSMs also ensures strong robustness in the arriving and sliding stages and weakens the risk of high gain. The simulation and experiment results show that the proposed control method is more effective than the traditional SMC, which has better rapidity, robustness, and anti-load-disturbance ability.

## Figures and Tables

**Table 1:** Parameters of GTUSM-60-R.

Symbol	Value	Symbol	Value
$R_{w}$	27.58 mm^2^	$I_{M}$	2 × 10^−5^ kg·m^2^
$I_{r}$	1.72 × 10^−5^ kg·m^2^	$C_{m}$	2.2 × 10^−4^ N·m·s
$C_{r}$	2.46 × 10^−4^ N·m·s	$C_{M}$	2.6 × 10^−4^ N·m·s
$f_{r}$	0.01 N·m·s	$f_{m}$	0.008 N·m·s
$I_{m}$	1.4 × 10^−5^ kg·m^2^	$f_{M}$	0.02 N·m·s

## Data Availability

Not applicable.
